# Risk Polymorphisms of *FNDC5*, *BDNF*, and *NTRK2* and Poor Education Interact and Aggravate Age-Related Cognitive Decline

**DOI:** 10.3390/ijms242417210

**Published:** 2023-12-07

**Authors:** Alessandra Mendonça Tomás, Natáli Valim Oliver Bento-Torres, Naina Yuki Vieira Jardim, Patrícia Martins Moraes, Victor Oliveira da Costa, Antônio Conde Modesto, André Salim Khayat, João Bento-Torres, Cristovam Wanderley Picanço-Diniz

**Affiliations:** 1 Neurodegeneration and Infection Research Laboratory, Institute of Biological Science, João de Barros Barreto University Hospital, Federal University of Pará, Belém 66073-000, Brazil; alessandratomas@ufpa.br (A.M.T.); naina.jardim@gmail.com (N.Y.V.J.); patriciamartins16@yahoo.com.br (P.M.M.); violiveiradacosta@gmail.com (V.O.d.C.); bentotorres@gmail.com (J.B.-T.); cwpdiniz@gmail.com (C.W.P.-D.); 2 Department of Physical Education, Federal University of Pará Application School, Belém 66095-780, Brazil; 3 Graduate Program in Human Movement Sciences, Federal University of Pará, Belém 66095-780, Brazil; 4 Graduate Program in Neuroscience and Cell Biology, Federal University of Pará, Belém 66050-160, Brazil; 5 Oncology Research Center (NPO), Graduate Program in Oncology and Medical Sciences, Federal University of Pará, Belém 66073-000, Brazil; antoniocmodesto@gmail.com (A.C.M.); khayatas@gmail.com (A.S.K.)

**Keywords:** risk factors, education, aging, polymorphism, genetic, cognition, memory, neuropsychological tests, neuroscience, primary prevention

## Abstract

Cognitive abilities tend to decline with aging, with variation between individuals, and many studies seek to identify genetic biomarkers that more accurately anticipate risks related to pathological aging. We investigated the influence of *BDNF*, *NTRK2*, and *FNDC5* single nucleotide polymorphisms (SNPs) on the cognitive performance of young and older adults with contrasting educational backgrounds. We addressed three questions: (1) Is education associated with reduced age-related cognitive decline? (2) Does the presence of SNPs explain the variation in cognitive performance observed late in life? (3) Is education differentially associated with cognition based on the presence of *BDNF*, *NTRK2*, or *FNDC5* polymorphisms? We measured the cognitive functions of young and older participants, with lower and higher education, using specific and sensitive tests of the Cambridge Automated Neuropsychological Test Assessment Battery. A three-way ANOVA revealed that SNPs were associated with differential performances in executive functions, episodic memory, sustained attention, mental and motor response speed, and visual recognition memory and that higher educational levels improved the affected cognitive functions. The results revealed that distinct SNPs affect cognition late in life differentially, suggesting their utility as potential biomarkers and emphasizing the importance of cognitive stimulation that advanced education early in life provides.

## 1. Introduction

The maintenance of cognitive functions is essential for functional ability in aging, and a shift from intervention to prevention is mandatory for the sustainability of human populations, which are currently facing a substantial demographic transition [1,2]. Indeed, recent epidemiological studies estimate that more than 55 million people will face dementia by 2050, compromising the quality of life of affected patients and their families and threatening economies at all scales—individual, familial, and global [3]. Neurodegenerative diseases that affect the cognitive performance of older adults, when they get worse, compromise their autonomy to carry out activities of daily living, increasing the annual costs for dementia-related assistance by about five times [4,5].

In line with this scenario, many studies have searched for biomarkers that more accurately anticipate the multifactorial risks related to pathological aging, providing individualized preventive actions [6]. Although out of the scope of this study, evidence-based information on brain dysfunction at the molecular, cellular, systemic, and behavioral scales, associating risk factors and corresponding preventive actions, is available elsewhere and may be useful to policymakers [7,8,9,10,11,12,13].

With the advent of new methodologies dedicated to examining events at a molecular scale, the contribution of genes to the identification of other non-modifiable risk factors for age-related cognitive decline has become apparent, and among them are the single nucleotide polymorphisms (SNPs) [14,15]. The genetics underlying cognitive ability and its relationship to age-related cognitive decline in healthy people became accessible and have been the subject of frequent studies over the last decade [16,17,18].

Formal education has a close and profound relationship with individual and collective health throughout life [19]. Pooling the evidence, it became clear that the longer the duration of formal education, the higher the levels of cognitive ability achieved [20]. In line with these studies, we demonstrated, using a transversal approach, a close relationship between the number of years of study early in life and the cognitive performance of older adults, with the higher education participants demonstrating greater performances in the neuropsychological assessment for attention, reaction time, learning, and memory [21].

A longitudinal study covering the age group from 35 to 74 years old, carried out with 14,594 healthy Brazilian adults, demonstrated that education seems to act more intensely than age when comparing its influences on cognitive performance [22].

Cognitive skills tend, however, to decline with age, with significant variation between individuals [23]. In fact, some seem to cope better with the brain changes imposed by aging while maintaining cognitive performance, while others quickly evolve into mild and severe cognitive declines [23,24,25]. The largely unexplained discrepancy between the amount of brain neuropathological changes and the degree of cognitive decline or functional impairment between individuals late in life [26] is at the root of the concept of cognitive reserve [27,28].

Among possible explanations for this unexplained discrepancy are the differential age-related synaptic loss and associated cognitive decline after reduced gene expression of brain-derived neurotrophic factor (*BDNF*) and its neurotrophic receptor tyrosine kinase 2—*NTRK2* (also known as *TRKB*) activity, which recapitulates the age-like pattern of expression markers of GABA inhibitory presynaptic genes [29]. Similarly, the cleaved circulating form of the exercise-induced membrane protein fibronectin type III domain containing 5—*FNDC5* (also known as irisin), released in the bloodstream following voluntary regular exercise, attenuates age-related cognitive dysfunction [30], and the cognitive impairment after genetic deletion of *FNDC5* in knockout mice can be rescued by irisin delivery directly into the dentate gyrus [31].

We selected two genes, *BDNF* and *NTRK2*, which have traditionally been linked to changes in cognitive functions and cognitive reserve based on the presence or absence of the risk allele [32,33]. Additionally, we chose *FNDC5*, a precursor of irisin, which plays a crucial role in metabolic functions and has shown reduced expression levels in Alzheimer’s disease [34,35]. Irisin, a myokine regulated by physical exercise, is involved in the development of brown adipose tissue and the transformation of white adipose tissue into a more metabolically active form.

Variations in *BDNF* and *NTRK2* polymorphisms have been associated with changes in hippocampal volume and improved performance in reasoning and memory tasks, depending on the specific nature of their polymorphisms [16,36,37]. Conversely, polymorphisms in the *FNDC5* gene have been linked to other pathologies, particularly diseases related to physical inactivity such as obesity, diabetes, and metabolic syndrome [38,39,40]. These diseases are known to be associated with poorer cognitive performance and, in more severe cases, dementia [41,42].

Thus, in the present report, we addressed three simple questions related to age-related cognitive decline: (1) Is education associated with reduced age-related cognitive decline? (2) Does the presence of single nucleotide risk polymorphisms of *BDNF*, *NTRK2*, or *FNDC5* explain at least part of the variation in cognitive performances observed late in life? (3) Is educational level differentially associated with cognition based on the presence of *BDNF*, *NTRK2*, or *FNDC5* polymorphisms?

## 2. Results

This section may be divided into subheadings. It should provide a concise and precise description of the experimental results, their interpretation, and the experimental conclusions that can be drawn.

When configuring the data analysis to measure the influence of age, education, and polymorphisms, we used two blocks of datasets based on the availability of information about SNPs. Thus, in the first group where the genotypic tests were not considered, there was a much larger number of subjects (*n* = 402) than in the group where the identification of SNPs was performed (*n* = 200). In both groups, we carried out a cross-sectional neuropsychological assessment, grouping individuals according to age and education in the first case and according to age, education, and polymorphism in the second case.

Despite discriminating the groups into lower and higher education, it is important to highlight right away that the average years of study of the two groups with lower education (YLE vs. OLE), as well as the average of the groups with higher education (YHE vs. OHE), are different from each other (*p* ≤ 0.001), and this is because 50% of the Brazilian population over 60 years of age has only primary education and only 26% complete secondary education, while the youngest, on average, remain in school until they finish high school, according to the School Census of the National Institute for Educational Studies and Research Anísio Teixeira—INEP [43]. This fact implies that in our sample, there is no difference in the average education of the YLE and OHE groups (*p* = 0.13). Although this discrepancy requires caution when interpreting the results between age groups, analyses within the same age group allow the use of education as a proxy (indicator) of cognitive reserve.

With these limitations in mind, we tested the hypothesis that young and older adult participants with higher education would present better cognitive performance in relation to peers with lower education. For this, we compared the groups using a two-way ANOVA, adopting the Bonferroni post hoc and the Cohen effect size (d).

### 2.1. Influence of Single Nucleotide Polymorphisms on Cognitive Performance

*BDNF* (rs6265), *NTRK2* (rs2289656), and *FNDC5* (rs3480) polymorphisms were identified using the blood samples collected from individuals in the previously established four groups. For detailed information on SNPs and the number of individuals in each group, see Table 1.

**Figure 1:**
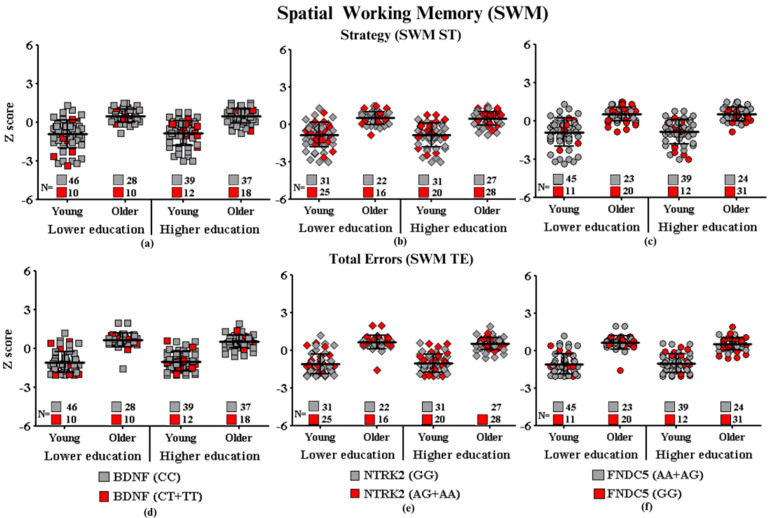
Spatial working memory performance according to gene polymorphisms: (**a**) strategy and *BDNF*, (**b**) strategy and *NTRK2*, (**c**) strategy and *FNDC5*, (**d**) total errors and *BDNF*, (**e**) total errors and *NTRK2*, and (**f**) total errors and *FNDC5*. Values are presented as the mean Z score and standard deviation.

**Figure 2:**
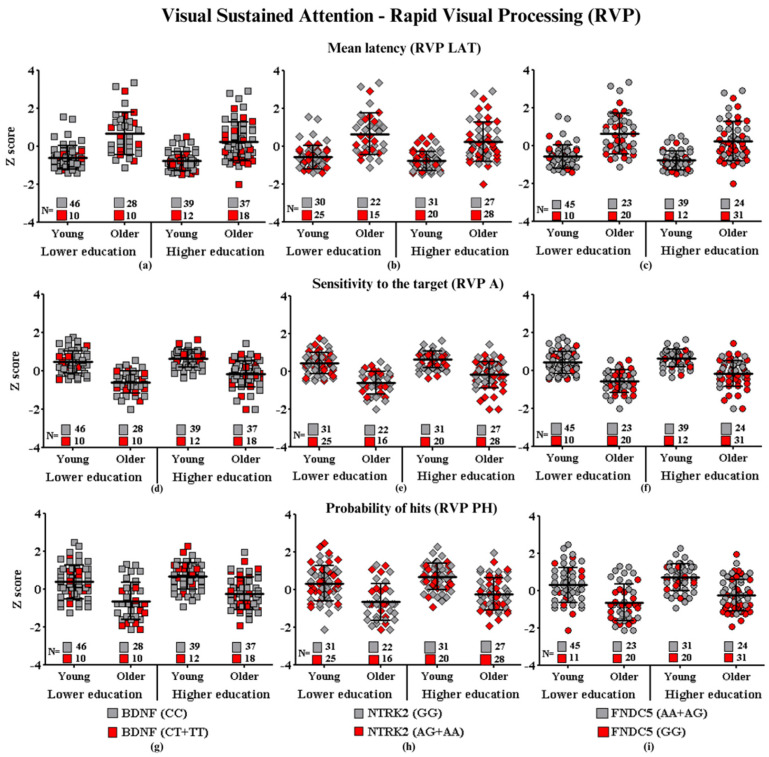
Visual Sustained Attention performance according to gene polymorphisms: (**a**) Mean latency and *BDNF*, (**b**) Mean latency and *NTRK2*, (**c**) Mean latency and *FNDC5*, (**d**) Sensitivity to the target and *BDNF*, (**e**) Sensitivity to the target and *NTRK2*, (**f**) Sensitivity to the target and *FNDC5*, (**g**) Probability of hits and *BDNF*, (**h**) Probability of hits and *NTRK2*, and (**i**) Probability of hits and *FNDC5*. Values are presented as the mean Z score and standard deviation.

**Figure 3:**
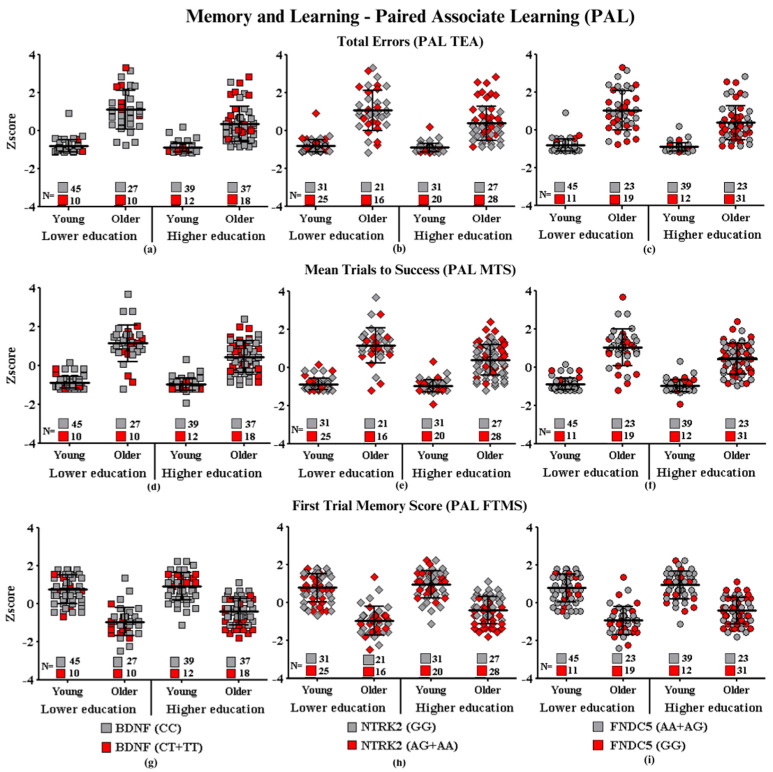
Learning and memory performance according to gene polymorphisms: (**a**) total error adjusted and *BDNF*, (**b**) total error adjusted and *NTRK2*, (**c**) total error adjusted and *FNDC5*, (**d**) mean trials to success and *BDNF*, (**e**) mean trials to success and *NTRK2*, (**f**) mean trials to success and *FNDC5*, (**g**) probability of hits and *BDNF*, (**h**) probability of hits and *NTRK2*, and (**i**) probability of hits and *FNDC5*. Values are presented as the mean Z score and standard deviation.

**Figure 4:**
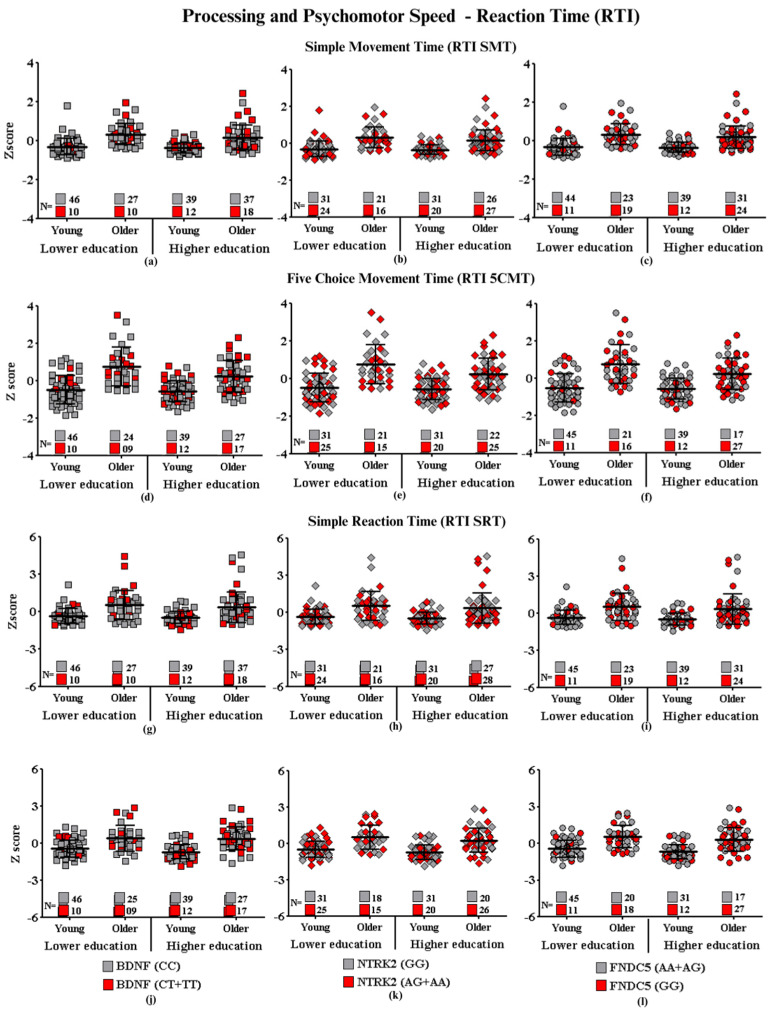
Processing and psychomotor speed performance according to gene polymorphisms: (**a**) Simple movement time and *BDNF*, (**b**) simple movement time and *NTRK2*, (**c**) simple movement time and *FNDC5*, (**d**) five-choice movement time and *BDNF*, (**e**) five-choice movement time and *NTRK2*, (**f**) five-choice movement time and *FNDC5*, (**g**) simple reaction time and *BDNF*, (**h**) simple reaction time and *NTRK2;* (**i**) simple reaction time and *FNDC5*, (**j**) five-choice reaction time and *BDNF*; (**k**) five-choice reaction time and *NTRK2*, and (**l**) five-choice reaction time and *FNDC5.* Values are presented as the mean Z score and standard deviation.

**Figure 5:**
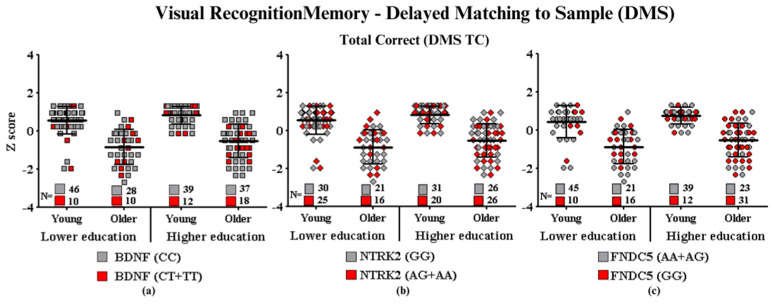
Visual recognition memory performance according to gene polymorphisms: (**a**) total correct and *BDNF*, (**b**) total correct and *NTRK2*, and (**c**) total correct and *FNDC5*. Values are presented as the mean Z score and standard deviation.

Graphic representations of cognitive performances in CANTAB tests (SWM, RVP, PAL, RTI, and DMS) (SWM: Spatial Working Memory; RVP: Rapid Visual Information Processing; PAL: Paired Associate Learning; RTI: Reaction Time; DMS: Delayed Match to Sample) of each group of participants carrying the SNPs of interest are shown in Figure 1, Figure 2, Figure 3, Figure 4 and Figure 5, which correspond, respectively, to SWM (Figure 1), RVP (Figure 2), PAL (Figure 3), RTI (Figure 4), and DMS (Figure 5). All figures show associations between the cognitive performances of each group on each CANTAB test and the polymorphisms associated with high and low risk for cognitive decline.

### 2.2. Age Affects Spatial Working Memory

Working memory allows the individual to temporarily store information necessary to continue a specific task, requiring the retention and manipulation of visuospatial information. In the evaluation of spatial working memory (SWM), the main effects of age were observed, both in the SWM strategy (SWM ST) (F_(3.398)_ = 270.60; *p* ≤ 0.0001) and in the total number of SWM total errors (SWM TE) (F_(3.398)_ = 503.41; *p* ≤ 0.0001). There was no influence of education (SWM ST: *p* = 0.70; SWM TE: *p* = 0.62) and no interaction between education and age (SWM ST: *p* = 0.16; SWM TE: *p* = 0.54) for this cognitive domain.

Comparisons between groups showed that for the SWM ST, the Young Lower Education group had better performance when compared to the Older Adults Lower Education (*p* ≤ 0.0001). Likewise, the Young Higher Education group performed better in the SWM ST compared to the Older Higher Education group (*p* ≤ 0.0001). Regarding the total number of errors on SWM, YLE performed better when compared to OLE (*p* ≤ 0.0001). Similarly, YHE made fewer mistakes compared to OHE (*p* ≤ 0.0001).

The results of the spatial working memory assessment revealed that aging is the main variable associated with significant decline. The SWM results for each group are shown in Table 2. The contribution of aging to the decline in spatial working memory can readily be identified by the strategy (d = 1.49, 1.91) and total error (d > 2) scores, where very large and enormous effect sizes emerge from the comparisons between young and old groups.

#### Spatial Working Memory

Potential associations between cognitive performances in the spatial working memory performance of the carriers of different polymorphisms and the genotypes of participants for *BDNF*, *NTRK2*, and *FNDC5* are shown in Figure 1.

**Subtitle: In the figure above, they are identified with “**■**”: the subjects with the highest risk in each group, and with “**■**”: the subjects with the lowest risk in each group. Each icon represents a sample subject. The black lines between the icons represent the means for each group. Squares are used to represent the *BDNF* gene, diamonds for the *NTRK2* gene, and circles for the *FNDC5* gene. On the left side of each graph are the groups with lower schooling (Young and Older), and on the right side are the groups with higher schooling (Young and Older). At the bottom of each graph, the number of participants in each group and their respective polymorphisms are highlighted.**

No main effects or interactions were observed for education, age, and *BDNF* (SWM ST: F_(7.192)_ = 0.59, *p* = 0.44; SWM TE: F_(7.192)_ = 0.16; *p* = 0.68), *NTRK2* (SWM ST: F_(7.192)_ = 0.001, *p* = 0.97; SWM TE: F_(7.192)_ = 1.29, *p* = 0.25) or *FNDC5* (SWM ST: F_(7.192)_ = 2.71, *p* = 0.10; and SWM TE: F_(7.192)_ = 0.003, *p* = 0.95) polymorphisms on cognitive performance. Despite the dispersion of the results on *BDNF*, the young participants have greater performance dispersion compared to the older adults, regardless of education. However, in all groups, the performance of participants at higher risk (CT + TT) was similarly distributed, with half of the participants performing below average and the other half above average, except for the YHE group, which has more participants with a higher risk above average (9/12) in SWM ST and below average (7/12) in SWM TE. *NTRK2* revealed a greater concentration of individuals with AG or AA *NTRK2* genotypes of higher risk SNPs around the mean and above it, which corresponds to a low-performance SWM strategy. The distribution of *FNDC5* results shows that the groups with higher education had more individuals with the GG genotype with better performance than the groups with lower education, reinforcing the possible neuroprotection of those with higher education. The number of participants with better performance (below the average) ranged from 5 out of 20 (5/20) for the OLE group, 10 out of 31 (10/31) in the OHE, 2 out of 11 (2/11) in the YLE, and 4 out of 12 (4/12) for the YHE (Figure 1a,b).

Figure 1 shows that aging in isolation seems to be a determinant for worse performances in spatial working memory and that education improves strategy scores in both the *NTRK2* and *FNDC5* polymorphism subgroups of young and older adults.

### 2.3. For Sustained Visual Attention, the More Years of Study the Better the Cognitive Performance

Both age and education influenced all measures of visual sustained attention performance (RVP). In the mean latency (RVP Lat), the main effects of age (F_(3.396)_ = 168.86; *p* ≤ 0.0001) and education (F_(3.396)_ = 9.85; *p* ≤ 0.002) were detected. Similarly, both target sensitivity (RVP A’) (age: F_(3.396)_ = 104.92; *p* ≤ 0.0001, and education: F_(3.396)_ = 14.10; *p* ≤ 0.0001) and the probability of correct answers (RVP PH) revealed main effects (age: F_(3.398)_ = 98.84; *p* ≤ 0.0001, and education: F_(3.398)_ = 13.61; *p* ≤ 0.0001), suggesting a significant influence of these variables on the probability of correct answers. However, the interaction between these measures did not show significant effects in any of the analyses (RVP latency: *p* = 0.74; RVP A: *p* = 0.26; and RVP PH: *p* = 0.51).

Comparisons between groups showed that YLE had a shorter latency time when compared to OLE (*p* ≤ 0.0001). The YHE group was more efficient in relation to the OHE (*p* ≤ 0.0001). However, the results of YLE vs. YHE were not statistically different (*p* = 0.07). When comparing OLE vs. OHE, OHE showed better performance in the group with higher education (*p* ≤ 0.005), which was more agile in identifying the target sequence and pressing the button in the RVP task.

In RVP A, which detects target sensitivity, it was observed that YLE performed better when compared to OLE (*p* ≤ 0.0001). Likewise, the young group with higher education had a better performance than the older adult group with higher education (*p* ≤ 0.0001). The YLE vs. YHE did not indicate differences between groups (*p* = 0.09). However, for the older adult groups (OLE vs. OHE), significant differences were observed, which suggests that the higher education group (OHE) was more sensitive in target perception (*p* ≤ 0.0001).

The probability of correct answers (RVP PH) showed significant differences when comparing YLE vs. OLE (*p* ≤ 0.0001). Likewise, the YHE group showed greater results when compared to the OHE (*p* ≤ 0.0001). Thus, younger people were more likely to answer the task correctly than older adults, regardless of their education level. When comparing the low vs. high scholarly, significant differences were observed for both young (*p* ≤ 0.006) and older adults (*p* ≤ 0.01), with groups with higher education performing better than groups with lower education.

Table 3 reveals the results for RVP, showing the values of the confidence interval and significant effect sizes. The effect size observed for RVP latency was considered very large (d = 1.37) for the influence of age and small for education (d = 0.31), suggesting that the average time required (in milliseconds) for the participants to adequately respond to the stimulus is affected in greater proportion by age.

#### Visual Sustained Attention—Rapid Visual Processing

Figure 2 shows the results for the effects of age, education, polymorphisms, and their possible interactions on visual sustained attention performance. Probability of hits (RVP PH), but not latency (RVP LAT) and sensitivity to the target (RVP A), showed main effects for age and *BDNF* (RVP LAT: F_(7.190)_ = 1.47, *p* = 0.22; RVP A: F_(7.192)_ = 0.22; *p* = 0.63; RVP PH: F_(7.192)_ = 4.05; *p* = 0.04). For *NTRK2*, RVP PH and RVP A showed main effects on the interaction between education and SNP (RVP PH: F_(7.192)_ = 4.02; *p* ≤ 0.04; RVP A: F_(7.192)_ = 6.66; *p* ≤ 0.01), but not on RVP LAT (F_(7.190)_ = 0.86, *p* = 0.35). For irisin/*FNDC5*, none of the variables showed main effects in the interaction with the polymorphisms (RVP LAT: F_(7.190)_ = 0.20, *p* = 0.64; RVP PH: F_(7.192)_ = 0.16; *p* = 0.41; RVP A: F_(7.192)_ = 1.00; *p* = 0.31). However no significant differences were observed in *post-hoc* Bonferroni analysis, suggesting that the presence or absence of the higher risk polymorphism does not influence the performance of the carriers of any of the investigated polymorphisms.

The descriptive analysis of the performance dispersion revealed that part of the measurements showed balanced performance dispersion, regardless of the polymorphism. For *BDNF*, for example, in the RVP LAT, the YLE CT + TT group (with the highest risk) had seven participants with average performance (7/10). In both OLE (5/10) and OHE (5/18), five participants performed above the average latency time, which represents a worse result. For both *NTRK2* and *FNDC5*, we observed that CT + TT older adults performed below average and younger adults performed on average. The other results were equivalent and therefore not highlighted.

**Subtitle: In the figure above, they are identified with “**■**”: the subjects with the highest risk in each group, and with “**■**”: the subjects with the lowest risk in each group. Each icon represents a sample subject. The black lines between the icons represent the means for each group. Squares are used to represent the *BDNF* gene, diamonds for the *NTRK2* gene, and circles for the *FNDC5* gene. On the left side of each graph are the groups with lower schooling (young and older), and on the right side are the groups with higher schooling (young and older). At the bottom of each graph, the number of participants in each group and their respective polymorphisms are highlighted.**

### 2.4. Age and Higher Education Impact on Memory and Learning Performance

The paired associative learning (PAL) of the CANTAB battery assesses the learning and memory performances and functions of the frontal and temporal lobes, specifically the associated paired learning and the visuospatial memory of the present time that depends on the functional integrity of the hippocampus [44].

In paired associative learning (PAL), age, education, and the interaction between them showed significant main effects on cognitive performances. For counting the total number of adjusted errors, all measures showed main effects (age: F_(3.396)_ = 268.72; *p* ≤ 0.0001; education: F_(3.396)_ = 10.93; *p* ≤ 0.001; and interaction: F_(3.396)_ = 4.09; *p* ≤ 0.04). Both the mean number of attempts to succeed (PAL MTS) and the number of successes in the first attempt (PAL FTMS) were influenced by age (PAL MTS: F_(3.396)_ = 349.64; *p* ≤ 0.0001; PAL FTMS: F_(3.396)_ = 286.87; *p* ≤ 0.0001) and education (PAL MTS: F_(3.396)_ = 10.37; *p* ≤ 0.001; PAL FTMS: F_(3.396)_ = 10.67; *p* ≤ 0.001). However, the interaction in these measures did not influence the performance (PAL MTS: *p* ≤ 0.09; PAL FTMS: *p* ≤ 0.31).

In the PAL TEA, the young group with lower education showed better results when compared with the OLE (*p* ≤ 0.0001). Likewise, the YHE group performed better when compared to the OHE (*p* ≤ 0.0001). The youth groups were not different from each other (*p* ≤ 0.41). Unlike young people, education influenced the results in groups of older adults who showed significant differences in this task (OLE vs. OHE: *p* ≤ 0.0001), with the group with higher education showing better performance than the group with low education.

The YLE group performed better than the OLE group in the PAL MTS (*p* ≤ 0.0001). However, education did not influence the results when comparing YLE and YHE, with no significant differences between their performances (*p* = 0.33). YHE showed better results in the task when compared to the older adult group with higher education (*p* ≤ 0.0001). OHE performed better than OLE (*p* ≤ 0.0001).

In the PAL FTMS, the performance of the young groups when compared to older adult groups with the same education was better (YLE vs. OLE: *p* ≤ 0.0001; YHE vs. OHE: *p* ≤ 0.0001). Young people were not different when compared to each other (*p* = 0.15). However, in older adults, education significantly influenced the results, with better performance in the OHE group (*p* ≤ 0.001) in relation to the OLE. The detailed results are shown in Table 4. Even though higher levels of education demonstrated better performance in the paired associative learning tests, it is important to note that in all comparative measures, the size effect was greater for age compared to educational level.

Our findings demonstrate that education seems to confer a neuroprotective effect on the older adult group. In fact, when young adults from different educational backgrounds are tested on the PAL, the performances are not significantly different, while older adults with higher education show better performances than those with low education in all forms of measuring paired associative learning. We found small effect sizes for education (d < 0.49) and very large and enormous effects on performance in the PAL test (d = 1.52, 2.00).

#### Learning and Memory—Paired Associated Learning

Paired associated learning assesses spatial and object recognition memory. To measure this function, we analyzed three different outcomes: (1) measures of adjusted errors—PAL TEA (informs the total number of errors, with an adjustment for each stage of the test, where higher scores reflect worse performances with a greater number of errors), (2) average number of attempts for success—PAL MTS (calculates the total number of attempts to find all patterns correctly in the tested stages and divides the result by the number of successfully completed steps), and (3) finding patterns on the first attempt—PAL FTMS (the number of patterns located correctly in the first attempt, added between the completed stages of the test, where higher indexes represent better performances) were used. The three variables (age, education, and polymorphisms) in the assessment of learning and memory (PAL test) interact only with *NTRK2*. In the PAL TEA, main effects in the three-way ANOVA were observed for *BDNF* and age (F_(7.199)_ = 4.60; *p* ≤ 0.03); *NTRK2* and education (F_(7.198)_ = 8.38; *p* ≤ 0.004); and *NTRK2*, education, and age (F_(7.198)_ = 11.54; *p* ≤ 0.001).

The intragroup analysis showed that older adults with *BDNF* genotypes associated with higher risk (CT + TT: 1.28 ± 0.37 Z score) performed worse than those with lower risk genotypes (CC: 1.09 ± 0.18 Z score) (*p* ≤ 0.007, 95% CI: 0.116, 0.729, d = 0.24). For the *NTRK2*, when comparing people with lower and higher education in a function of their SNPs risk, differences were observed only in the group with higher education, with participants with the highest risk SNP (0.14 ± 0.16 Z score) performing worse than the lowest risk (−0.51 ± 0.07 Z score) (*p* ≤ 0.0001, 95% CI: 0.47, 1.008, d = 0.80).

Similarly, it was identified that the older adults with higher education who presented the *NTRK2* higher risk polymorphism (AG + AA: 0.86 ± 0.18 Z score) performed worse when compared to the subgroup with the lower risk (GG: −0.07 ± 0.10 Z score). The other comparisons were not significant in the post-hoc analysis. Figure 3a shows that both *BDNF* and *NTRK2* present a greater number of OHE participants with a higher risk of performing above average, implying worse performances than the lower risk subgroup of these two genes. The *FNDC5* gene did not show differences in any of the investigations.

Regarding measurement of the PAL MTS, the main effects in the three-way ANOVA were observed for *BDNF* and age (F_(7.199)_ = 4.18; *p* ≤ 0.04); *NTRK2* and education (F_(7.198)_ = 12.99; *p* ≤ 0.0001); and *NTRK2*, education, and age (F_(7.198)_ = 14.78; *p* ≤ 0.0001).

Differences in *BDNF* (*p* ≤ 0.01, 95% CI: 0.08, 0.65, d = 0.80) were only observed in OHE, with the higher risk genotypes participants (CT + TT: 0.97 ± 0.17 Z score) performing worse than those with lower risk genotypes (CC: 0.64 ± 0.11 Z score).

High-educated participants with *NTRK2* SNP (*p* ≤ 0.001, 95% CI: 0.15, 0.61, d = 0.85) with higher risk genotypes (AG + AA: 0.09 ± 0.15 Z score) showed worse performances when compared with participants with lower risk genotypes (GG: −0.48 ± 0.09 Z score). Indeed, higher-educated participants with AG + AA required more attempts to complete the task correctly than those with SNP GG. In the same way, only in the OHE, differences were observed between those with the high-risk genotype (0.83 ± 0.14 Z score) in relation to those with a lower-risk genotype (0.06 ± 0.12 Z score). *FNDC5* did not differ in subgroup analyses of their polymorphisms on PAL MTS measures.

Similarly, in the task of finding patterns on the first attempt (PAL FTMS), main effects in the three-way ANOVA were observed for *BDNF* and age (F_(7.192)_ = 6.76; *p* ≤ 0.01); *NTRK2* and age (F_(7.191)_ = 5.51; *p* ≤ 0.02); *NTRK2*, education, and age (F_(7.191)_ = 4.91; *p* ≤ 0.02); and *FNDC5* and age (F_(7.192)_ = 5.49; *p* ≤ 0.02).

For *BDNF* and age (*p* ≤ 0.002, 95% CI: −0.84,−0.18, d = 0.79), only the older adults showed significant results in the comparisons of high-risk polymorphisms (−0.96 ± 0.13 Z score) and low-risk polymorphisms (−0.46 ± 0.09 Z score); likewise, *NTRK2* (*p* ≤ 0.001, 95% CI: −0.78, −0.19, d = 1.55) just older adults group had difference between high risk (AA + AG: −0.89 ± 0.10 Z score) and low risk (GG:−0.37 ± 0.11 Z score) polymorphisms. In the interaction between *NTRK2*, age, and education, just OHE *NTRK2* showed differences (*p* ≤ 0.0001, 95% CI: −1.13, −0.59, d = 0.80), and OHE high risk (AA + AG: −0.82 ± 0.11 Z score) had the worst performance when compared to OHE low risk (AA + AG: 0.03 ± 0.10 Z score). In both measures, the higher-risk polymorphisms presented worse results.

*FNDC5* and age interact (*p* ≤ 0.01, 95% CI: 0.09, 0.76, d = 0.40). Post-hoc analysis showed that high-risk polymorphisms negatively affect the performance of young adults on PAL FTMS but not that of older adults. Young adults with higher risk showed a lower performance (GG: 0.79 ± 0.10 Z score) when compared to young adults with lower risk (AG + AA: 0.95 ± 0.10 Z score).

**Subtitle: In the figure above, they are identified with “**■**”: the subjects with the highest risk in each group, and with “**■**”: the subjects with the lowest risk in each group. Each icon represents a sample subject. The black lines between the icons represent the means for each group. Squares are used to represent the *BDNF* gene, diamonds for the *NTRK2* gene, and circles for the *FNDC5* gene. On the left side of each graph are the groups with lower schooling (young and older), and on the right side are the groups with higher schooling (young and older). At the bottom of each graph, the number of participants in each group and their respective polymorphisms are highlighted.**

### 2.5. Reaction Time Increases with Aging Regardless of Education

RTI assesses motor and mental response speeds, as well as measures of movement time, reaction time, and response accuracy. Psychomotor speed, a skill that relates cognition to physical movement, indicates the individual’s ability to detect, perceive, and respond to a given stimulus. Frontoparietal circuits are activated by this, so that worse performance in RTI tasks may indicate reduced white matter integrity, especially during the aging process, compromising independence from daily life activities.

The main effects of age increased the reaction time (RTI) measured as a Simple Accuracy Score (SAS): RTI SAS: F_(3.396)_ = 24.87; *p* ≤ 0.0001). Both education (*p* = 0.25) and the interaction between age and education (*p* = 0.50) were not significant. Likewise, the measurement of reaction time performed with the five-choice modality revealed a significant influence of age on reaction time (RTI 5CAS: F_(3.396)_ = 10.73; *p* = 0.001), but education or interaction showed no statistical significance (education: *p* = 0.14; interaction: *p* = 0.39).

For RTI SAS and RTI 5CAS, the YLE group performed better than the OLE in the simple task (RTI SAS: *p* ≤ 0.002), but not in the task with five choices (RTI 5CAS: *p* = 0.08). The YHE group was better than the OHE, both in the single-choice task (*p* ≤ 0.0001) and in the five-choice task (*p* = 0.004). Scholarly age groups did not influence performances in both YLE vs. YHE (RTI SAS: *p* = 0.25; RTI 5CAS: *p* = 0.14) and OLE vs. OHE (RTI SAS: *p* = 0.70; RTI 5CAS: *p* = 0.62). These results are shown in Table 5.

When movement time is measured in the simple choice mode (Simple Movement Time—SMT) only age showed the main effects (RTI SMT: F_(3.396)_ = 22.93; *p* ≤ 0.0001), while education (*p* = 0.90) or interaction (*p* = 0.97) were not significant. In the five-choice stage (Five Choice Movement Time—5CMT), both age (RTI 5CMT: F_(3.374)_ = 89.47; *p* ≤ 0.0001) and education (RTI 5CMT: F_(3.374)_ = 9.23; *p* ≤ 0.003) were significant, but the interaction between them did not influence the performance (*p* = 0.09).

In the RTI SMT and RTI 5CMT movement time tasks, young groups were faster than older adult groups, regardless of education. YLE was better than OLE (RTI SMT: *p* ≤ 0.001; RTI 5CMT: *p* ≤ 0.0001). Likewise, YHE was more efficient than OHE (SMT RTI: *p* ≤ 0.001; 5CMT RTI: *p* ≤ 0.0001). However, YLE vs. YHE were not different in both analyses (*p* = 0.92 and *p* = 0.38, respectively). In the simple task, the older adult groups were not different from each other (*p* = 0.94); however, in the five-choice task, OLE was slower than OHE (*p* ≤ 0.0001).

In simple response time, age showed the main effects on cognitive performance (RTI SRT: F_(3.396)_ = 37.09; *p* ≤ 0.0001), while education (*p* = 0.49) or interaction (*p* = 0.32) did not influence the results. Likewise, for the RTI 5CRT, only age (F_(3.374)_ = 25.58; *p* ≤ 0.0001) had a significant main effect, while education (*p* = 0.07) and interaction (*p* = 0.30) did not influence the results.

In the simple response time (RTI SRT) and with five choices (RTI 5CRT), the young groups in OLE responded faster when compared to the older adult groups, regardless of education. Thus, YLE was faster than OLE in simple tasks (*p* ≤ 0.0001) and with five choices (*p* ≤ 0.0001). YHE was OLE more responsive than OHE on both measures (RTI SMT: *p* ≤ 0.0001; RTI 5CRT: *p* ≤ 0.0001). YLE did not differ from more educated peers (YHE) (RTI SRT: *p* = 0.29; RTI 5CRT: *p* = 0.06). Similarly, the older adults were not different from each other in any of the estimates (RTI SRT: *p* = 0.80; RTI 5CRT: *p* = 0.53). All results are shown in Table 6.

Taken together, the Reaction Time (RTI) test results, measured as reaction time and movement time, suggest that motor and mental response rates, as well as movement time, reaction time, and acuity of response, are affected by aging. Only in the movement time task with five choices, there was a neuroprotective effect of education on cognition during the aging process. Education did not affect the younger participants’ performances. We found large effect sizes for the aging effects (0.86 ≤ d ≤ 1.01) and small effects (0.21 ≤ d ≤ 0.44) on performance in the RTI.

#### Processing and Psychomotor Speed—Reaction Time

No main effects were observed in the three-way ANOVA for *BDNF* (RTI SMT: F_(7.190)_ = 1.20, *p* = 0.27; RTI 5CMT: F_(7.185)_ = 2.22, *p* = 0.14), *NTRK2* (RTI SMT: F_(7.190)_ = 1.20, *p* = 0.27; RTI 5CMT: F_(7.185)_ = 0.83, *p* = 0.36), and *FNDC5* (RTI SMT: F_(7.190)_ = 0.58, *p* = 0.44; RTI 5CMT: F_(7.185)_ = 3.62, *p* = 0.06) regarding movement time (Figure 4a) or response times in *BDNF* (RTI SRT: F_(7.190)_ = 0.001, *p* = 0.97; RTI 5CRT: F_(7.174)_ = 0.73, *p* = 0.39), *NTRK2* (RTI SRT: F_(7.190)_ = 0.19, *p* = 0.65; RTI 5CRT: F_(7.174)_ = 3.15, *p* = 0.07), or *FNDC5* (RTI SRT: F_(7.190)_ = 0.26, *p* = 0.60, RTI 5CRT: F_(7.174)_ = 0.08, *p* = 0.76) (Figure 4b), regardless of the gene, age, or education. The main effects were only detected between *NTRK2* and education on five-choice response time (RTI 5CRT: F_(7.174)_ = 10.27, *p* = 0.002). Only on participants with lower education, the post-hoc analyses indicated significantly worse performance of the participants with the highest risk SNP (AG + AA: 0.14 ± 0.16 Z score) than participants with the lowest risk (GG: −0.51 ± 0.07 Z score) (*p* ≤ 0.01, 95% CI: 0.10, 0.78, d = 0.23).

Figure 4d shows that despite no main effects related to the *NTRK2* gene, it is possible to observe different distributions of performances for the OLE participants: among the fifteen subjects who present higher risk polymorphism, only 3 in 15 subjects (3/15) showed below-average performance, which in general indicates that the higher risk group was slower in detecting and responding to the stimulus than peers carrying lower risk polymorphism.

**Subtitle: In the figure above, they are identified with “**■**”: the subjects with the highest risk in each group, and with “**■**”: the subjects with the lowest risk in each group. Each icon represents a sample subject. The black lines between the icons represent the means for each group. Squares are used to represent the *BDNF* gene, diamonds for the *NTRK2* gene, and circles for the *FNDC5* gene. On the left side of each graph are the groups with lower schooling (young and older), and on the right side are the groups with higher schooling (young and older). At the bottom of each graph, the number of participants in each group and their respective polymorphisms are highlighted.**

The psychomotor measures assessed through this test establish a relationship between cognitive response and movement, which integrates perception and speed response. From comparative results, it emerged that five-choice tasks are more sensitive to identifying subtle changes than single-choice tasks.

### 2.6. Recognition Memory Performance Worsens with Age and Improves with Education

Finally, the analysis of pairing and short-term delayed visuospatial recognition (DMS) indicated the presence of main effects in the probability of errors after correct answers for both age (DMS PEGC: F_(3.313)_ = 150.03; *p* ≤ 0.0001) and education (DMS PEGC: F_(3.313)_ = 4.24; *p* ≤ 0.05). Main effects were also identified in the measure of the probability of error after error for age (DMS PEGE: F_(3.267)_ = 43.76; *p* ≤ 0.0001) and education (DMS PEGE: F_(3.267)_ = 5.16; *p* ≤ 0.05), as well as the measurement of the total number of correct answers for age (DMS TC: F_(3.314)_ = 196.00; *p* ≤ 0.0001) and education (DMS TC: F_(3.314)_ = 7.67; *p* ≤ 0.005). However, we did not find the main effects of the interaction between education and age (DMS PEGC: *p* = 0.24; DMS PEGE: *p* = 0.75; DMS TC: *p* = 0.33).

The post-hoc results are compiled in Table 6 and indicate that the YLE showed a lower probability of errors after correct answers (DMS PEGC) and after errors (DMS PEGE) as compared to the OLE (DMS PEGC: *p* ≤ 0.0001; DMS PEGE: *p* ≤ 0.0001). YHE was OLE less likely to make mistakes compared to OHE (DMS PEGC: *p* ≤ 0.0001; DMS PEGE: *p* ≤ 0.0001). YLE vs. YHE were different in the probability of error after success (*p* ≤ 0.05), but not in the probability of error after error (*p* = 0.21). The older adult groups were not different from each other in the DMS PEGC (*p* = 0.51), but they were different in the DMS PEGE (*p* ≤ 0.05).

Regarding the total number of correct answers (DMS TC), both YLE and YHE had better performances compared to the older adult groups. YLE got more correct task stages than OLE (*p* ≤ 0.0001). Likewise, YHE correctly responded more than OHE (*p* ≤ 0.0001). YHE got more correct responses than YLE (*p* ≤ 0.01). However, OLE vs. OHE showed no significant differences (*p* = 0.18).

The size of the effects varied according to the task (0.30 ≤ d ≤ 1.71) for the probability of error after the correct answer (DMS PEGC) and total number of correct attempts (DMS TC), with a minor influence of education as compared to age. Detailed information is available in Table 7.

#### Visual Recognition Memory—Delayed Matched to the Sample

Object recognition memory is the main cognitive function assessed in the Delayed Matching to Sample test (DMS). Through it, it is possible to measure the performance of the participants through the total number of correct answers (DMS TC), that is, by considering the number of attempts in which the participant was able to discriminate between the colors and shapes of a standard image, respecting the time interval between pattern exposure and response possibilities.

No main effects were observed in the three-way ANOVA for any of the investigated genes. The comparison of higher risk subgroups vs. lower risk polymorphisms of the *BDNF* (F_(7.189)_ = 0.25, *p* = 0.61), *NTRK2* (F_(7.189)_ = 1.74, *p* = 0.18), and *FNDC5* (F_(7.189)_ = 2.34, *p* = 0.12) genes did not reveal significant differences that would suggest the influence of SNPs for this function, which may suggest that recognition memory does not seem to be affected by these polymorphisms. In Figure 5, we recognize that the groups showed a homogeneous distribution, regardless of the age and education of the subjects.

**Subtitle: In the figure above, they are identified with “**■**”: the subjects with the highest risk in each group, and with “**■**”: the subjects with the lowest risk in each group. Each icon represents a sample subject. The black lines between the icons represent the means for each group. Squares are used to represent the *BDNF* gene, diamonds for the *NTRK2* gene, and circles for the *FNDC5* gene. On the left side of each graph are the groups with lower schooling (young and older), and on the right side are the groups with higher schooling (young and older). At the bottom of each graph, the number of participants in each group and their respective polymorphisms are highlighted.**

## 3. Discussion

In this study, we investigated the potential influence of single nucleotide polymorphisms on the cognitive performance of young and older adults with contrasting educational backgrounds. For these tests, we measured executive functions through the spatial working memory function and test that requires activation of the frontal cortex [45]; the Rapid Visual Information Processing and Reaction Time tests that assess sustained attention and visual processing and psychomotor speed requiring the activation of fronto-striatal circuits [46,47]; the Paired Associative Learning test, which assesses the ability to learn and episodic memory, being dependent on the integrity of networks in the medial temporal lobe (hippocampus) and frontal cortices [48,49]; and finally, the Delayed matching to sample evaluate both visual matching ability and visual recognition memory [50,51].

The analyses of these functions confirmed and expanded our previous results using the same battery of neuropsychological tests [21]. It was demonstrated that the education of the older adult groups influenced the performance of specific functions such as sustained attention, paired associative learning, short-term object recognition memory, and processing speed.

Consistently, age had a significant influence on cognitive performance, regardless of the presence of risk polymorphisms. Paired associative learning was the function most affected by the investigated polymorphisms. The presence of the risk polymorphism of *BDNF* and *NTRK2* affected the performance in the three evaluated measures: total errors adjusted (PAL TEA), mean trials to success (PAL MTS), and first trial memory score (PAL FTMS), while the polymorphism of *FNDC5* influenced only the measures of the first trial memory score (PAL FTMS). The results obtained for paired associative learning were identified in the interaction of *BDNF* with age (PAL TEA, PAL MTS, and PAL FTMS), *NTRK2* with age and education (PAL TEA, PAL MTS, and PAL FTMS), as well as *NTRK2* separately with education (PAL TEA and PAL MTS) and with age (PAL FTMS), whereas *FNDC5* showed interaction with age only for PAL FTMS.

### 3.1. Education, Cognitive Reserve, and Aging

Age-related cognitive decline affects multiple domains with appreciable individual variability [24]. Some individuals preserve their cognitive functions until the end of life, revealing healthy aging, while others show cognitive deterioration rapidly as they age [52]. It has been demonstrated that education and occupational activities providing continuous multisensory and cognitive stimulation are associated with a lower risk of dementia [53,54,55]. A longitudinal study covering the age group from 35 to 74 years old, carried out with 14,594 healthy adults in Brazil, demonstrated that education seems to act more intensely than age when comparing its influences on cognitive performance [22]. Cross-sectional studies have confirmed the contribution of education to preserving executive functions during aging [21] and that this relative protection is associated with greater cognitive reserve [56,57].

Many functional associations have been made between the cognitive reserve of older adults and the preservation of cognitive functions, including executive functions, learning, and episodic memory [58], with robust evidence that in the healthy aging population, education has a neuroprotective effect by slowing the progression of cognitive decline in individuals with higher education [56,59]. Our findings, when taken together, confirm a number of previous studies indicating that aging promotes multidimensional cognitive decline, influencing in a variable way the various cognitive domains tested. In opposition to senile cognitive decline, education improved the performance of measures of rapid visual information processing (RVP), associated paired learning (PAL), delayed matching to sample (DMS), and reaction time measured through the five-choice modality (RTI 5CMT). In addition, we detected that age and education interact in the paired learning task measured by the total number of adjusted errors (PAL TEA), influencing performance in episodic memory tests.

Most studies involving cognitive reserve are based on “proxies” (indirect indicators that reflect the variable of interest) presumably associated with expanded cognitive reserve, such as education, occupational activities, and entertainment [60,61]. There seems to be a close association between education, cognitive reserve, and cognitive performance in a healthy older adult population [62], and it has been suggested that education and reading ability can be used as proxies of cognitive reserve [63].

Thus, adopting education as an indicator (proxy) of cognitive reserve [53,64], and the fact that the older adults with higher education in the present report revealed better performance in the PAL, RVP, DMS, RTI, and 5CMT tests, it is reasonable to assume that these individuals acquired greater cognitive reserve and neuroprotection for these functions than those with lower education.

The cognitive reserve has multifactorial individual aspects beyond the educational level, such as occupation complexity, performance of mentally stimulating activities, marital status during middle age, the level of activity and physical exercise, and support and social engagement in old age [65,66]. In the presence of these individual aspects, older adults with low educational levels showed better global cognitive performance, especially older women. Furthermore, high cognitive reserve capacity throughout life attenuates the impacts of smaller hippocampal volumes and reinforces the compensatory effect of high cognitive reserve in neurodegenerative diseases [66]. Purpose in life promotes a reduction in the neuropathological effects observed in middle-aged individuals and, therefore, a positive impact on brain reserve and cognitive function [67]. In addition, physical activity associated with cognitive stimulation during leisure time contributes to the maintenance of cognitive reserve and has a protective impact on brain health [65]. The additive effect of multifactorial individual aspects reduces the risk of neurodegenerative diseases [68].

In this context, a limitation of our study is not to include other important factors in cognitive reserve. However, the educational level is considered the main proxy of the cognitive reserve [53,64] and has been recurrently associated with better cognitive performance and a lower risk for the development of dementia [69]. Once the educational level is directly linked to economic condition, the complexity of work, and social engagement in old age [70].

### 3.2. Genetic Polymorphisms Education and Senile Cognitive Decline

#### 3.2.1. Val66Met (rs6265) of *BDNF*

Brain-derived neurotrophic factor (*BDNF*) is a member of the most abundant neurotrophins family in the mammalian brain, contributing to the differentiation of neuronal populations in the central and peripheral nervous systems and synaptic plasticity during development and in adult life [71,72]. *BDNF* expression is controlled by the *BDNF* gene found at locus 11p14.1 [73], contributing to numerous cognitive functions, including those related to learning and long-term visual memory [74].

A recent investigative effort devoted to *BDNF* variants has shown that while none of the *BDNF* variants, including their “anti-sense” transcripts (*BDNF*-AS, anti-sense transcript), also called anti-*BDNF* [73], showed any significant influence on visual working memory, three variants were significantly associated with long-term visual memory [74].

Frois et al. (2022), in a more recent study, also found negative results for the influence of the Val66Met polymorphism (rs6265) of *BDNF* on executive functions [75], while tests for evaluation of long-term memory confirmed the presence of a significant interaction of that variant with long-term visual mnemonic decline [76]. In line with these analyses, the present study investigated this association in individuals with contrasting ages and education and found an influence of *BDNF* polymorphisms on the performance in the visual working memory test.

In the *BDNF* polymorphism clusters, it was possible to detect age influences on performance for all Paired Associative Learning (PAL) tasks. The intragroup comparison revealed that older adult participants with the highest-risk genotype (CT + TT) presented worse performances in all PAL measures when compared to older adult participants with lower risk genotypes (CC), regardless of their education. In contrast, no interaction was observed with education.

We observed that episodic memory proved to be more sensitive to age than to the individual’s level of education. The risk variants (CT + TT) were shown to be associated with important functional changes, which are clearly caused by the aging process, such as smaller hippocampal volumes [77], reduced *BDNF* secretion, and worse performance in episodic memory [78,79], as well as increased susceptibility to the development of Alzheimer’s disease [80].

Similar to our findings, Egan et al. [78] indicated that young adults who have the highest risk variants (CT + TT) of *BDNF* perform worse on memory tasks when compared to lower risk variants (CC). Furthermore, Kennedy e cols. [80] found that age and *BDNF* polymorphisms explain 27% of the variation in performance in associative memory tasks, demonstrating the important effect of age on the memory of individuals aged approximately 55 years. However, there is no consensus in the literature, and previous studies failed to find the same results in the presence of higher risk variants of *BDNF* [81,82,83].

#### 3.2.2. *NTRK2* (rs2289656)

The *NTRK2* gene (neurotrophic tyrosine receptor kinase 2) encodes one of the members of the family of neurotrophic receptors linked to the cell membrane that, after interaction with *BDNF*, maintains cognitive health [29,84].

Here, we demonstrated that education, age, and polymorphisms interact and alter performance in the paired associative learning tests for all forms of PAL measurement (PAL TEA, PAL MTS, and PAL FTMS). Significant interactions were only observed between education and age and between education and polymorphisms, influencing the results for PAL TEA and PAL MTS, but only for age and polymorphism in the case of PAL FTMS. Thus, high education in association with lower risk genotypes generates better neuroprotection for episodic memory. In line with our observations, *BDNF* and its receptor play a key role in long-term potentiation (LTP) and dendritic development, which are essential parts of the formation of episodic memory [85].

In contrast, the comparison of higher risk vs. lower risk subgroups of *NTRK2* gene polymorphisms did not reveal significant differences between performances in the DMS task, which may suggest that short-term pattern recognition memory does not seem to be influenced by this group of polymorphisms.

Five-choice reaction time (RTI 5CRT) analyses revealed that groups with low education with AA + AG polymorphisms (higher risk) presented worse performance compared to those with low education with GG polymorphisms (lower risk).

#### 3.2.3. *FNDC5* (rs3840)

The *FNDC5* gene (a short form of “fibronectin type III domain containing 5”) encodes the irisin protein that is released after cleavage of the transmembrane precursor protein that is expressed in muscle. Irisin is a myokine released into the circulation during physical exercise that stimulates *BDNF* expression in the hippocampus [86]. The central administration of irisin modulates the expression of genes related to neuroplasticity in the hippocampus and the prefrontal cortex [87]. Furthermore, irisin deficiency induced in models for Alzheimer’s disease employing knockout mice compromises long-term potentiation and object recognition memory, while its peripheral overexpression restores memory deficits [35].

Irisin/*FNDC5* is not only involved in metabolic functions and linked to conditions such as diabetes, obesity, and cerebrovascular disease [34,88] but also plays a crucial role in mediating the benefits of physical exercise [89]. This holds true for both healthy older adult individuals and in models simulating Alzheimer’s disease, where its expression is diminished [35]. Notably, the increase in irisin triggers the production of neurotrophic factors in the hippocampus, a pivotal region for memory formation [35,86]. The rs3480 SNP of *FNDC5* encodes the irisin precursor protein and can impact the expression and activity of irisin, thereby influencing the body’s response to physical exercise [90]. In broader terms, the G allele has been associated with an elevated risk of changes in glucose metabolism and heightened susceptibility to type 2 diabetes mellitus due to reduced irisin expression [88,91]. Given the compelling evidence demonstrating a close connection between diabetes and Alzheimer’s disease [41,42,92], and the fact that age is the most significant risk factor for Alzheimer`s disease, we have explored the potential impact of these polymorphisms on cognitive function in healthy older adults.

Here, we found the main effects on the episodic memory test (PAL FTMS), in which age and SNP significantly interact. The post-hoc analyses revealed that for the PAL FTMS, the young people with a higher risk SNP (GG) performed worse than the young people with a lower risk SNP (AG + AA). However, we do not know how to explain why this variation in performance is limited to young people, and this requires further studies. None of the other neuropsychological tests, such as SWM, RVP, RTI, and DMS, were influenced by *FNDC5* polymorphisms.

This result seems counterintuitive if it were not for the fact that a previous study, dedicated to investigating potential associations between performance in executive function tests and irisin, revealed a negative modulatory action of irisin on executive functions, with impairment of cognitive flexibility and inhibitory response [93]. Actually, from a neurophysiological point of view, it has been suggested that the contribution of irisin to the modulation of executive functions may occur through GABAergic neurons and *BDNF* [94]. The presence of irisin in the nervous system has been detected in neurons immunostained for GABA-amino-butyric acid, whose dysfunction seems to originate from cognitive decline in humans, particularly that related to Alzheimer’s disease [95]. Similarly, hippocampal *BDNF* induction through the *FNDC5* pathway favors long-term potentiation and object recognition memory [35] but does not influence executive functions [75].

The ability to acquire and maintain focus on a given stimulus or task requires sustained attention, and this is an essential part of the cognitive tasks that support activities of daily living [96]. With aging, sustained attention decreases, and this becomes apparent in cognitive tests that assess it [97].

### 3.3. Limitations and Future Directions

Automated tests, such as those of the CANTAB battery used in this study, reduce the time and costs of their application, increase the ease with which data are collected using automated scoring and normative comparisons, offer accuracy in the millisecond scale for response times, allow the individualized collection of the components of a given task, guarantee the standardization of the procedures, and allow the evaluation of multiple cognitive domains [98]. The CANTAB battery, however, does not seem to discriminate discretely against cognitive functions by measuring cognitive domains in healthy older adults that eventually do not exactly match those of the standardized neuropsychological assessment batteries [49].

In fact, for Robbins and collaborators, four distinct functions can be identified in the CANTAB battery: learning and memory, response speed, executive functions, and perceptual ability [99]. Of all the tests applied in the CANTAB battery, the Associated Paired Learning (PAL) was the only one that effectively measured what it proposed to measure and analyzed the same functions as the standardized visual episodic memory tests. Thus, except for PAL results, data from other tests (DMS, RTI, RVP, and SWM) cannot be directly compared with those from standardized tests, limiting the comparability of results [49]. For these authors, the RVP, for example, tests multidimensional cognitive domains that include all the factors contained in the CANTAB battery, while the classic standardized tests allow the discrete analysis of the components.

Another limitation was related to the fact that the combination of more than one polymorphism in the same individuals can effectively change the molecular scenario, combining actions that can change the phenotype of the carriers. It has already been shown, for example, that when investigating the association of the single nucleotide polymorphism (rs6265) of *BDNF*, combined with other polymorphisms, including *APOE* (rs429358), *KIBRA* (rs17070145), and *CLSTN2* (rs6439886), the decline in episodic memory was accelerated. Such a research possibility stimulates new research projects on combined polymorphisms to explain the molecular mechanisms associated with senile cognitive decline [100]. This inheritance of combined polymorphisms was not the object of this study and, therefore, requires significant additional effort in the future to adapt resources and sample size.

The investigation of simultaneous interactions between age, education, and SNPs in relation to the PAL MTS performances of young and older groups with varying levels of education and *NTRK2* polymorphisms was hindered by the limited sample size. To address this limitation, we employed the bootstrapping technique as a statistical procedure for random resampling. Interestingly, the three-way ANOVA analysis of *NTRK2* polymorphisms across all groups yielded significant differences in the results obtained with and without resampling, particularly in samples with higher variability, such as those of older adults. This discrepancy underscores the need for further investigation and caution in interpreting these findings. For a more comprehensive understanding, please refer to Supplementary Tables S1 and S2.

## 4. Materials and Methods

Before the research program started, the local Research Ethics Committee of the University Hospital João de Barros Barreto (CAEE n° 25946814.4.0000.0017) approved all procedures, and all ethical recommendations in research involving human subjects were observed. Participants voluntarily signed the consent forms before performing the procedures.

### 4.1. Participants

We performed a cross-sectional neuropsychological evaluation of 402 individuals grouped as older adults (*n* = 252) and young adults (*n* = 150). Both groups were organized as a function of their years of education early in life, as depicted in Table 8.

All individuals had no history of head trauma, stroke, primary depression, or chronic alcoholism. All participants had normal cognitive performance as assessed in the Mini-Mental State Examination (MMSE), adjusted for education level according to the criteria for the Brazilian population with the following cutoffs: illiterate, 13 points; 1–7 years of education, 18 points; and ≥8 years of education, 26 points [101]. All patients who met these criteria realized anamnesis, followed by neuropsychological assessment using selected tests of the Cambridge Automated Neuropsychological Assessment Battery—CANTAB. All individuals had a blood sample collected.

### 4.2. Neuropsychological Assessment

The MMSE and CANTAB tests were administered by trained investigators in an appropriate environment with adequate lighting and reduced noise. The MMSE results were recorded with pencil and paper and required verbal interaction with the investigators. Responses for CANTAB tests used a touchscreen and were largely independent of verbal instructions. All participants were assessed individually. We started with the motor screening task (MOT), which introduced the CANTAB touchscreen to the individuals. The MOT detects whether sensorimotor or other difficulties are related to the collection of valid data from each participant. After touchscreen adaptation, we selected the following CANTAB tests: (1) Spatial Working Memory (SWM) for measures of the retention and manipulation of visuospatial information; (2) Rapid Visual Information Processing (RVP), which is sensitive to sustained attention; (3) Paired Associate Learning (PAL) to assess visual memory and new learning; (4) Reaction Time (RTI) that provides assays of motor and mental response speeds, as well as measures of movement time, reaction time and response accuracy; (5) Delayed Matching to Sample (DMS) assesses forced-choice recognition memory for visual patterns and tests both simultaneous matching and short-term visual memory. The detailed information about CANTAB is available elsewhere [21,102,103].

### 4.3. DNA Extraction, Quantification, and Dilution

For DNA extraction, 500 microliters of the total peripheral blood of the patients was used, as previously described in the “Salting Out” protocol as follows: red blood cell lysis, leukocyte lysis, protein precipitation, and DNA elution. The DNA of each sample was quantified using the SpectraDrop—Micro Volume Starter Kit (Molecular Devices), with an absorbance index of 260 nm. Samples were then diluted at a concentration of 10 ng/µL for later analysis by polymerase chain reaction (PCR).

### 4.4. Analysis of Single Nucleotide Polymorphisms (SNPs)

Three SNP markers associated with neurotrophins and cognition were selected, as shown in Table 9.

For identification of the SNPs referring to the *BDNF* (rs6265), *NTRK2* (rs2289656), and irisin/*FNDC5* (rs3480) genes, a DNA concentration of 10 ng/µL was used to be processed in the RT-PCR 7300 Applied Biosystems. For real-time PCR (Life Technologies, Carlsbad, CA, USA), TaqMan^®^ probes were used (Applied Biosystems, Foster City, CA, USA) marked with VIC/FAM fluorophores, and the samples were genotyped according to the manufacturer’s recommendations. Optimized reagents had a final volume of approximately 8 µL, consisting of 3.5 µL of Master Mix, 0.175 µL of Taqman^®^ probe, 3.325 µL of water, and 1.0 µL of sample DNA. The final mix was amplified with the following schedule: 2 min at 50 °C, 10 min at 95 °C, 40 cycles of 15 s at 95 °C, and 1 min at 60 °C. Once the genotyping was completed, a qualitative analysis of the results was carried out, which were exported and evaluated individually for all probes using the 7300 real-time PCR system (Applied Biosystems).

### 4.5. Statistical Analysis

Outlier values (±3 SD from the mean) were removed prior to statistical analysis. Next, the assumptions of normality and homogeneity of the sample were evaluated using the Shapiro–Wilk and Levene tests, respectively. Considering the distribution and to standardize the different comparison scales between the outcome measures, cognitive test performance was transformed into Z scores. The *p* values associated with these statistical tests were set to ≥0.05, which indicates that the ANOVA was appropriate in the present analysis.

We used two-way ANOVA and Bonferroni post-hoc tests for analyses between education and age, where the results of cognitive tests associated with different levels of education (higher vs. lower level) were compared. The four groups formed according to age (20 to 40 years for young adults and ≥60 years for older adults) and the educational status (low and high) of the participants were compared, namely, young lower education—YLE (elementary and high school); young higher education—YHE (college education); older lower education—OLE (elementary school); older higher education—OHE (high school and college education).

Three-way analysis of variance (ANOVA) and Bonferroni post-hoc tests were used to investigate the effects of age, education, polymorphisms, and their possible interactions on the cognitive performances of young and older adults. To test the potential influence on cognitive performances of the polymorphisms of interest, we compared young and older adult groups with lower and higher education and their respective SNPs:*BDNF*-rs6265 (CC vs. CT + TT), *NTRK2*—rs22289656 (GG vs. AG + AA), and irisin/*FNDC5* (GG vs. AG + AA). For this hypothesis test, we standardized the sample using random resampling (bootstrapping for 5000 samples), which, according to previous studies, maintains the power of the statistical test if the initial sample indicates significance *p* ≤ 0.01 [104].

Significant statistical differences were plotted as mean and standard error (mean ± standard error). Categorical variables were represented by the number of subjects (n). The statistical significance level was established considering the value of *p* ≤ 0.05 (two-tailed), the confidence interval (95% CI), and the Cohen’s d effect size, based on the Z score, which was classified as very small (d = 0.01, 0.19), small (d = 0.20, 0.49), medium (d = 0.50, 0.79), large (d = 0.80, 1.1), very large (d = 1.2, 1.9), or enormous (d ≥ 2.0) [105]. All statistical analyses were performed using SPSS^®^ Statistics software (version 20; IBM Corporation, Armonk, NY, USA) and Graph Pad PRISM^®^ for graphic illustrations.

## 5. Conclusions

The results of the neuropsychological assessments in association with the polymorphisms found in groups of adults of contrasting ages and educations revealed that the single nucleotide polymorphisms for the investigated genes (*BDNF*, *NTRK2*, and *FNDC5*) differentially affect episodic memory.

Single nucleotide polymorphisms for *BDNF* Val66Met, *NTRK2*, and *FNDC5* differentially influenced the performance in episodic memory tests, with *BDNF* and *FNDC5* being related only to age and *NTRK2* associated with age and education, with carriers of the allele of risk demonstrating lower performance.

The level of education (cognitive reserve) has been shown to positively influence the cognitive performance of volunteers in tasks of sustained attention, psychomotor speed, recognition, and episodic memory. The benefits are more pronounced in sustained attention measured through rapid visual information processing (RVP) as well as in episodic memory measured by the paired learning test (PAL) in the older.

Thus, the response to the three addressed questions in the present report related to age-related cognitive decline and *BDNF*, *NTRK2*, or *FNDC5* risk polymorphisms is yes, they affect cognition late in life and may explain at least part of the variation in older adults’ cognitive performances, and yes, education improves affected functions.

## Figures and Tables

**Table 1:** Genotypic characteristics of the groups. Values presented as the number of participants.

Groups	Young Lower Education	Young Higher Education	Older Lower Education	Older Higher Education	Total
SNP *BDNF* (rs6265)	CC: 46 CT + TT: 10	CC: 39 CT + TT: 12	CC: 28 CT + TT: 10	CC: 37 CT + TT: 18	CC: 150 CT + TT: 50
SNP *NTRK2* (rs2289656)	GG: 31 AA + AG: 25	GG: 31 AA + AG: 20	GG: 22 AA + AG: 16	GG: 27 AA + AG: 28	GG: 111 AA + AG: 89
SNP *FNDC5* (rs3840)	GG: 11 AA + AG: 45	GG: 12 AA + AG: 39	GG: 16 AA + AG: 22	GG: 31 AA + AG: 24	GG: 70 AA + AG: 130

**Table 2:** Working memory—Spatial Working Memory (SWM) results. Values are shown as the mean Z score ± SD.

Tests	Groups (n)	Mean ± SD	CI 95%	d	Age (F)	Education (F)	Interaction (F)
SWM strategy (score)	Young lower education (75) Older lower education (126)	−0.75 ± 1.04 0.44 ± 0.61	−1.81, −1.17 ***	1.49	270.60 ***	0.14	1.91
	Young lower education (75) Young higher education (75)	−0.75 ± 1.04 −0.89 ± 0.97	−0.18, 0.46	0.14			
	Young higher education (75) Older higher education (126)	−0.89 ± 0.97 0.52 ± 0.56	1.57, −2.25 ***	1.91			
	Older lower education (126) Older higher education (126)	0.44 ± 0.61 0.52 ± 0.56	−0.11, 0.38	0.14			
SWM total errors (score)	Young lower education (75) Older lower education (126)	−0.97 ± 0.82 0.61 ± 0.61	1.91, 2.63 ***	2.27	503.41 ***	0.23	0.36
	Young lower education (75) Young higher education (75)	−0.97 ± 0.82 −0.96 ± 0.81	−0.31, 0.33	0.01			
	Young higher education (75) Older higher education (126)	−0.96 ± 0.81 0.53 ± 0.48	−2.76, −2.02 ***	2.39			
	Older lower education (126) Older higher education (126)	0.61 ± 0.61 0.53 ± 0.48	−0.10, 0.39	0.15			

***: *p* ≤ 0.001. CI: confidence interval; d: effect size—Cohen’s d.

**Table 3:** Visual sustained attention—Rapid visual information processing (RVP) results. Values are shown as the mean Z score ± SD.

Tests	Groups	Mean ± SD	CI 95%	d	Age (F)	Education (F)	Interaction (F)
RVP latency (ms)	Young lower education (75) Older lower education (126)	−0.58 ± 0.58 0.57 ± 0.96	1.05, 1.69 ***	1.37	168.86 ***	9.85 **	0.10
	Young lower education (75) Young higher education (75)	−0.58 ± 0.58 −0.82 ± 0.46	0.13, 0.78	0.46			
	Young higher education (75) Older higher education (126)	−0.82 ± 0.46 0.27 ± 0.96	1.03, 1.66 ***	1.34			
	Older lower education (126) Older higher education (126)	0.57 ± 0.96 0.27 ± 0.96	0.06, 0.56 **	0.31			
RVP A’ (score)	Young lower education (75) Older lower education (126)	0.46 ± 0.59 −0.57 ± 1.15	0.75, 1.35 ***	1.05	104.92 ***	14.10 ***	1.27
	Young lower education (75) Young higher education (75)	0.46 ± 0.59 0.69 ± 0.49	0.10, 0.75	0.42			
	Young higher education (75) Older higher education (126)	0.69 ± 0.49 −0.12 ± 0.86	1.32, 1.98 ***	1.65			
	Older lower education (126) Older higher education (126)	0.57 ± 1.15 −0.12 ± 0.86	0.19, 0.69 ***	0.44			
RVP probability of hits (score)	Young lower education (75) Older lower education (126)	0.37 ± 0.97 −0.47 ± 0.92	0.60, 1.19 ***	0.89	98.84 ***	13.61 ***	0.42
	Young lower education (75) Young higher education (75)	0.37 ± 0.97 0.76 ± 0.78	0.12, 0.77 **	0.44			
	Young higher education (75) Older higher education (126)	0.76 ± 0.78 −0.20 ± 0.84	0.87, 1.48 ***	1.17			
	Older lower education (126) Older higher education (126)	−0.47 ± 0.92 −0.20 ± 0.84	0.06, 0.55 **	0.31			

**: *p* ≤ 0.01; ***: *p* ≤ 0.001. CI: confidence interval; d: effect size—Cohen’s d.

**Table 4:** Learning and memory—Paired associates learning (PAL). Values are shown as the mean Z score± SD.

Tests	Groups	Mean ± SD	CI 95%	d	Age (F)	Education (F)	Interaction (F)
PAL total errors adjusted (score)	Young lower education (75) Older lower education (126)	−0.75 ± 0.34 0.69 ± 0.96	1.61, 2.39 ***	2.00	268.72 ***	10.93 **	4.09 *
	Young lower education (75) Young higher education (75)	−0.75 ± 0.34 −0.85 ± 0.24	0.02, 0.66	0.34			
	Young higher education (75) Older higher education (126)	−0.85 ± 0.24 0.27 ± 0.91	1.20, 1.84 ***	1.52			
	Older lower education (126) Older higher education (126)	0.69 ± 0.96 0.27 ± 0.91	0.20, 0.70 ***	0.45			
PAL mean trials to success (score)	Young lower education (75) Older lower education (126)	−0.81 ± 0.40 0.71 ± 0.87	1.25, 1.90 ***	1.57	349.64 ***	10.37 ***	2.83
	Young lower education (75) Young higher education (75)	−0.81 ± 0.40 −0.93 ± 0.31	0.01, 0.66	0.34			
	Young higher education (75) Older higher education (126)	−0.93 ± 0.31 0.34 ± 0.87	1.44, 2.11 ***	1.78			
	Older lower education (126) Older higher education (126)	0.71 ± 0.87 0.34 ± 0.87	0.18, 0.54 ***	0.42			
PAL first trial memory score (score)	Young lower education (75) Older lower education (126)	0.73 ± 0.75 −0.66 ± 0.74	1.53, 2.21 ***	1.87	286.87 ***	10.67 ***	0.99
	Young lower education (75) Young higher education (75)	0.73 ± 0.75 0.91 ± 0.80	−0.09, 0.55	0.23			
	Young higher education (75) Older higher education (126)	0.91 ± 0.80 −0.32 ± 0.73	1.30, 1.95 ***	1.63			
	Older lower education (126) Older higher education (126)	−0.66 ± 0.74 −0.32 ± 0.73	0.21, 0.71 ***	0.46			

*: *p* ≤ 0.05; **: *p* ≤ 0.01; ***: *p* ≤ 0.001. CI: confidence interval; d: effect size—Cohen’s d.

**Table 5:** Accuracy—Reaction time (RTI). Values are shown as the mean Z score ± SD.

Tests	Groups	Mean ± SD	CI 95%	d	Age (F)	Education (F)	Interaction (F)
RTI simple accuracy score (score)	Young lower education (75) Older lower education (126)	0.22 ± 0.43 −0.21 ± 1.16	0.16, 0.74 **	0.45	24.87 ***	1.28	0.44
	Young lower education (75) Young higher education (75)	0.22 ± 0.43 0.40 ± 0.28	0.17, 0.82	0.50			
	Young higher education (75) Older higher education (126)	0.40 ± 0.28 −0.16 ± 1.22	0.28, 0.86 ***	0.57			
	Older lower education (126) Older higher education (126)	−0.21 ± 1.16 −0.16 ± 1.22	−0.20, 0.29	0.04			
RTI five-choice accuracy score (score)	Young lower education (75) Older lower education (126)	0.09 ± 0.70 −0.15 ± 1.29	−0.07, 0.50	0.22	10.73 ***	2.10	0.71
	Young lower education (75) Young higher education (75)	0.09 ± 0.70 0.32 ± 0.39	0.08, 0.73	0.41			
	Young higher education (75) Older higher education (126)	0.32 ± 0.39 −0.09 ± 1.02	0.23, 0.62 **	0.50			
	Older lower education (126) Older higher education (126)	−0.15 ± 1.29 −0.09 ± 1.02	−0.27, 0.37	0.05			

**: *p* ≤ 0.01; ***: *p* ≤ 0.001. CI: confidence interval; d: effect size—Cohen’s d.

**Table 6:** Processing and psychomotor speed—Reaction time (RTI). Values are shown as the mean Z score ± SD.

Tests	Groups	Mean ± SD	CI 95%	d	Age (F)	Education (F)	Interaction (F)
RTI simple movement time (ms)	Young lower education (75) Older lower education (126)	−0.29± 0.40 0.18± 0.48	0.74, 1.34	1.04	22.93 ***	0.01	0.001
	Young lower education (75) Young higher education (75)	−0.29± 0.40 −0.31 ± 0.29	−0.26, 0.38	0.06			
	Young higher education (75) Older higher education (126)	−0.31 ± 0.29 0.17± 1.62	0.08, 0.66	0.37			
	Older lower education (126) Older higher education (126)	0.18± 0.48 0.17± 1.62	−0.24, 0.26	0.01			
RTI five-choice movement time (ms)	Young lower education (75) Older lower education (126)	−0.47± 0.71 0.56± 1.05	0.79, 1.40	1.10	89.47 ***	9.23 **	2.79
	Young lower education (75) Young higher education (75)	−0.47± 0.71 −0.60 ± 0.54	−0.11, 0.53	0.21			
	Young higher education (75) Older higher education (126)	−0.60 ± 0.54 0.12 ± 0.97	0.56, 1.16	0.86			
	Older lower education (126) Older higher education (126)	0.56± 1.05 0.12 ± 0.97	0.19, 0.69	0.44			
RTI simple reaction time (ms)	Young lower education (75) Older lower education (126)	−0.29 ± 0.59 0.21± 1.00	−0.73, −0.28	0.58	37.09 ***	0.47	0.98
	Young lower education (75) Young higher education (75)	−0.29± 0.59 −0.46± 0.49	−0.01, 0.64	0.31			
	Young higher education (75) Older higher education (126)	−0.46± 0.49 0.24 ± 1.24	0.38, 0.96	0.67			
	Older lower education (126) Older higher education (126)	0.21± 1.00 0.24 ± 1.24	−0.22, 0.27	0.03			
RTI five-choice reaction time (ms)	Young lower education (75) Older lower education (126)	−0.36± 0.70 0.36 ± 1.06	0.47, 1.06	0.76	72.70 ***	3.27	
	Young lower education (75) Young higher education (75)	−0.36 ± 0.70 −0.63 ± 0.71	0.06, 0.71	0.38			
	Young higher education (75) Older higher education (126)	−0.63 ± 0.71 0.28 ± 0.97	0.73, 1.33	1.03			
	Older lower education (126) Older higher education (126)	0.36 ± 1.06 0.28 ± 0.97	−0.24, 0.40	0.08			

**: *p* ≤ 0.01; ***: *p* ≤ 0.001. CI: confidence interval; d: effect size—Cohen’s d.

**Table 7:** Visual recognition memory—Delayed matching to sample (DMS). Values are shown as the mean Z score ± SD.

Tests	Groups	Mean ± SD	CI 95%	d	Age (F)	Education (F)	Interaction (F)
DMS probability of error given correct (score)	Young lower education (75) Older lower education (126)	−0.45 ± 0.68 0.57 ± 1.04	0.80, 1.41 ***	1.11	150.03 ***	4.24 *	1.33
	Young lower education (75) Young higher education (75)	−0.45 ± 0.68 −0.74 ± 0.46	0.17, 0.82 *	0.50			
	Young higher education (75) Older higher education (126)	−0.74 ± 0.46 0.49 ± 0.92	1.25, 1.90 ***	1.57			
	Older lower education (126) Older higher education (126)	0.57 ± 1.04 0.49 ± 0.92	−0.17, 0.33	0.08			
DMS probability of error given error (score)	Young lower education (75) Older lower education (126)	−0.35 ± 0.86 0.45 ± 1.09	0.50, 1.09 ***	0.79	43.76 ***	5.16 *	0.09
	Young lower education (75) Young higher education (75)	−0.35 ± 0.86 −0.57 ± 0.62	−0.03, 0.62	0.29			
	Young higher education (75) Older higher education (126)	−0.57 ± 0.62 0.15 ± 0.93	0.57, 1.17 ***	0.87			
	Older lower education (126) Older higher education (126)	0.45 ± 1.09 0.15 ± 0.93	0.05, 0.54 *	0.30			
DMS total correct (score)	Young lower education (75) Older lower education (126)	0.48 ± 0.73 −0.65 ± 0.90	1.03, 1.66 ***	1.34	196.00 ***	7.67 **	0.93
	Young lower education (75) Young higher education (75)	0.48 ± 0.73 0.81 ± 0.49	0.21, 0.86 **	0.53			
	Young higher education (75) Older higher education (126)	0.81 ± 0.49 −0.49 ± 0.88	1.38, 2.04 ***	1.71			
	Older lower education (126) Older higher education (126)	−0.65 ± 0.90 −0.49 ± 0.88	−0.07, 0.43	0.18			

*: *p* ≤ 0.05; **: *p* ≤ 0.01; ***: *p* ≤ 0.001. CI: confidence interval; d: effect size—Cohen’s d.

**Table 8:** Age, sex, and education of participants. Values are shown as the mean ± standard error.

Groups	Young Lower Education (YLE)	Young Higher Education (YHE)	Older Lower Education (OLE)	Older Higher Education (OHE)
Participants (n)	75	75	126	126
Age (years)	25.49 ± 2.32	26.98 ± 2.12	70.79 ± 2.25	69.55 ± 2.35
Sex (n)	Female: 37 Male: 38	Female: 36 Male: 39	Female: 102 Male: 24	Female: 99 Male: 27
MMSE (points)	29.04 ± 1.60	29.23 ± 0.83	27.00 ± 2.27	28.32 ± 1.25
Education (years)	12.94 ± 1.60	17.66 ± 1.37	5.22 ± 1.38	12.14 ± 1.57

MMSE: Mini-Mental State Examination.

**Table 9:** Polymorphisms and associated outcomes.

Gene/rs	Vic/Fam Fluorophores	Localization	Outcomes
*BDNF*/rs6265	C/T	Chr.11: 27658369	T allele associated with memory impairment ^1^, increases susceptibility to developing Alzheimer’s disease ^2^. Associated with reduced hippocampal volume ^3^.
*NTRK2*/rs2289656	A/G	Chr.9: 84948647	G allele, lower risk of cognitive impairment ^4^. Patients with Alzheimer’s disease are more frequently heterozygous for this gene ^5^.
*FNDC5*/rs3480	A/G	Chr.1: 32862564	G allele associated with reduced expression, greater susceptibility to diabetes mellitus, and its metabolic dysfunctions ^6^.

^1^: Ng et al., 2019 [79]; ^2^: Kennedy et al., 2015 [80]; ^3^: Karnik et al., 2010 [77]; ^4^: Matyi et al., 2017; ^5^: Cozza et al., 2008; ^6^: Yang et al., 2022 [88]. *BDNF*: brain-derived neurotrophic factor; *NTRK2*: neurotrophic receptor tyrosine kinase 2; *FNDC5*: fibronectin type III domain containing 5.

## Data Availability

The data presented in this study are openly available at https://github.com/NataliBento-Torres/Tomas_Ageing-cognition-education-and-single-nucleotide-polymorphisms.git (accessed on 6 November 2023).

## Supplementary Materials

The following supporting information can be downloaded at: https://www.mdpi.com/article/10.3390/ijms242417210/s1.
