# Supplementary material for: Risk Polymorphisms of FNDC5, BDNF, and NTRK2 and Poor Education Interact and Aggravate Age-Related Cognitive Decline
Source: Int J Mol Sci. 2023 Dec 7;24(24):17210. doi: 10.3390/ijms242417210 (PMC10743741; doi:10.3390/ijms242417210)
Supplement: Supplementary file 1 [file ijms-24-17210-s001.zip › ijms-2699751-supplementary.pdf]

Risk Polymorphisms of *FNDC5, BDNF,* and *NTRK2* and Poor Education Interact and Aggravate Age-Related Cognitive Decline

Alessandra Mendonça Tomás [1,2], Natáli Valim Oliver Bento-Torres [1,3,*], Naina Yuki Vieira Jardim [1,4], Patrícia Martins Moraes [1,3], Victor Oliveira da Costa [1,4], Antônio Conde Modesto [5], André Salim Khayat [5], João Bento-Torres [1,3] and Cristovam Wanderley Picanço-Diniz [1,4,5]

Supplementary Table S1. Significant CANTAB results in three-way ANOVA. Values are shown as mean Z score ± SD.

Tests	Education	Age	SNP NTRK2 (n)	Mean ± SD	CI 95%	d
PAL total errors adjusted (score)	Lower	Young	AA+AG (25)	-0.73 ± 0.40	-0.23, 0.44	0.32
			GG (31)	-0.83 ± 0.22		
		Older	AA+AG (16)	1.01 ± 1.14	-0.64, 0.18	0.23
			GG (21)	1.24 ± 0.92		
	**Higher**	Young	AA+AG (20)	-0.86 ± 0.27	-0.34, 0.37	0.05
			GG (31)	-0.87 ± 0.18		
		**Older**	**AA+AG (28)**	**0.86 ± 0.97**	**0.59, 1.27*****	**1.19**
			**GG (27)**	**-0.07 ± 0.52**		
**PAL mean trials to success (score)**	**Lower**	Young	AA+AG (25)	-0.83 ± 0.32	-0.26, 0.35	0.16
			GG (31)	-0.88 ± 0.29		
		**Older**	**AA+AG (16)**	**0.87 ± 0.94**	**-0.88, -0.12****	**0.58**
			**GG (21)**	**1.38 ± 0.83**		
	**Higher**	Young	AA+AG (20)	-0.94 ± 0.40	-0.32, 0.33	0.03
			GG (31)	-0.95 ± 0.22		
		**Older**	**AA+AG (28)**	**0.83 ± 0.77**	**0.45, 1.07*****	**1.07**
			**GG (27)**	**0.06 ± 0.67**		
**PAL first trial memory score (score)**	Lower	Young	AA+AG (25)	0.74 ± 0.72	-0.46, 0.28	0.12
			GG (31)	0.83 ± 0.77		
		Older	AA+AG (16)	-1.02 ± 0.80	-0.57, 0.34	0.16
			GG (21)	-0.90 ± 0.72		
	**Higher**	Young	AA+AG (20)	0.99 ± 0.63	-0.32, 0.46	0.10
			GG (31)	0.92 ± 0.79		
		**Older**	**AA+AG (28)**	**-0.82 ± 0.57**	**-1.23, -0.48*****	**1.54**
			**GG (27)**	**0.03 ± 0.53**		

Significant results in bold. *: p≤0.05; **: p≤0.01; ***: p≤0.001. CI: confidence interval; d: effect size - Cohen's d. SNP: single nucleotide polymorphism.

Supplementary Table S2. Significant CANTAB results in three-way ANOVA with Bootstrapping. Values are shown as mean Z score ± SD.

Tests	Education	Age	SNP NTRK2 (rs2289656)	Mean ± SD	CI 95%	d
**With Bootstrapping 5000 samples**						
**PAL total errors adjusted (score)**	Lower	Young	AA+AG	-0.73 ± 0.40	-0.49, 0.29	0.32
			GG	-0.83 ± 0.22		
		Older	AA+AG	1.01 ± 1.14	-0.90, 0.44	0.23
			GG	1.24 ± 0.92		
	**Higher**	Young	AA+AG	-0.86 ± 0.27	-0.09, 0.14	0.05
			GG	-0.87 ± 0.18		
		**Older**	**AA+AG**	**0.86 ± 0.97**	**0.53, 1.37*** **	**1.19**
			**GG**	**-0.07 ± 0.52**		
**PAL mean trials to success (score)**	Lower	Young	AA+AG	-0.83 ± 0.32	-0.11, 0.21	0.16
			GG	-0.88 ± 0.29		
		Older	AA+AG	0.87 ± 0.94	-1.11, 0.07	0.58
			GG	1.38 ± 0.83		
	**Higher**	Young	AA+AG	-0.94 ± 0.40	-0.18, 0.20	0.03
			GG	-0.95 ± 0.22		
		**Older**	**AA+AG**	**0.83 ± 0.77**	**0.38, 1.15*** **	**1.07**
			**GG**	**0.06 ± 0.67**		
**PAL first trial memory score (score)**	Lower	Young	AA+AG	0.74 ± 0.72	-0.47, 0.30	0.12
			GG	0.83 ± 0.77		
		Older	AA+AG	-1.02 ± 0.80	-0.60, 0.39	0.16
			GG	-0.90 ± 0.72		
	**Higher**	Young	AA+AG	0.99 ± 0.63	-0.31, 045	0.10
			GG	0.92 ± 0.79		
		**Older**	**AA+AG**	**-0.82 ± 0.57**	**-1.13, -0.59*** **	**1.54**
			**GG**	**0.03 ± 0.53**		

Significant results in bold. *: p≤0.05; **: p≤0.01; ***: p≤0.001. CI: confidence interval; d: effect size - Cohen's d. SNP: single nucleotide polymorphism.
